# Letting go and saying goodbye: a Nepalese family’s decision, in the Ethiopian Airline crash ET-302

**DOI:** 10.1080/20961790.2021.1995945

**Published:** 2021-12-06

**Authors:** Samarika Dahal

**Affiliations:** Department of Oral Pathology and Forensic Dentistry, Tribhuvan University, Kathmandu, Nepal

Death is inevitable. Grief following a death is universal. In expressing the loss of loved ones, grief differs across cultures and individuals. It may vary depending upon the circumstances and the person’s ability to cope with the loss of their loved ones [1]. The probable potential return of a loved one vanishes in confirmed identification of the deceased. The experience is different in an unconfirmed death. “Ambiguous loss” was first coined by Pauline Boss, which results when families of missing persons have no clue of the whereabouts of their loved ones, whether they are either dead or alive, (or if they are dead), the location of the remains is unknown [2]. There are two fundamental types of ambiguous loss. In the first type, people are psychologically present but physically absent. Though presumed to be dead, their remains are never recovered. Consequently, the family members are engrossed with the missing person’s thoughts even years after the event. In the second type, people are alive and physically present but psychologically absent due to depression, addiction, or dementia. Both these conditions can coexist in the same family of the missing person [3, 4].

Different families react to ambiguous loss differently. Certain family members fail to overcome this despair and grief and are described as “living in limbo” spending their whole life in anticipation of their loved one’s arrival. Internal closure never occurs, and such families are never at peace and never move on. Conversely, other families move on and end the grief by taking robust decisions [5, 6]. This article highlights a case of an ambiguous loss in an Ethiopian Airlines flight crash. Following this incident, a Nepalese family bravely bided farewell to their loved one without waiting for her biological remains to be recovered. Such dialectical thinking of this family, I believe, is brave and defies conventional thinking. During such an incident, in my opinion, authorities should respect the family’s decision based on the informed particular options available to them without others telling them what they should do.

The Ethiopian Airlines flight ET-302, (a Boeing 737-MAX 8) crashed shortly after take-off from Addis Ababa Bole International Airport (HAAB) in Ethiopia on March 10, 2019, at about 05:44 UTC killing 149 passengers and seven crew members [7]. Ethiopia appointed International Criminal Police Organization (INTERPOL) to coordinate global Disaster Victim Identification (DVI) action relating to the crash. On March 12, an INTERPOL Incident Response Team (IRT) was deployed to Addis Ababa to coordinate the international police DVI response with Ethiopia. Ethiopian Airlines contracted Blake Emergency Services, a private company in the UK, to support the victim identification process. Ethiopia appointed the INTERPOL General Secretariat to manage the international Ante Mortem (AM) Data Reception Center. Continuous interaction in Addis Ababa between international agencies, embassies, and the INTERPOL IRT was coordinated to extract the AM records. Some countries did not provide the AM data for their deceased nationals. The INTERPOL General Secretariat followed up directly with these countries to obtain full AM records. A Nepalese citizen was one of the victims whose family did not provide any AM data. AM data including family DNA is essential for scientific identification.

The author conducted a telephone interview with a close family member on June 12, 2019. The Nepalese victim’s family declined to provide a DNA reference sample. However, they provided some relevant AM data, which was then communicated to the INTERPOL office of Nepal. They made a decision not to claim the human remains of their loved one, which was unconventional, but courageous in the author’s opinion. On asking, “under what circumstances was this decision taken”, they replied, “they are aware of the fact that her remains cannot be retrieved” and “funeral rituals according to the Hindu tradition shouldn’t wait”. Thus, they completed the funeral rituals with the charred soil collected from the crash site. It was their choice for a final goodbye.

Ethiopian Airlines flight ET-302 crashed, killing all passengers and crew members from 35 countries [8]. Nearly 100 DVI experts from 14 countries in Africa, the Americas and Europe supported the work of the IRT during its 50-day mission. INTERPOL centralized the collection of DNA and fingerprint materials from the families of the victims to aid in the identification process. All the dental AM data were also collected. Within 6 months of the tragedy, the DVI team positively identified every single victim [9]. However, the family of the Nepalese citizen decided not to claim the body of their loved one. The family decided to continue with the grief without the physical remains. It was a contentious decision. A few issues might disturb them in the coming days: would she be alive if she had taken a different route or had stayed longer? Also, the family might experience the shame of not being able to say goodbye.

In situations like this, people can be frozen and immobilized by ambiguous loss, which prevents them from beginning the mourning process [10]. In the absence of physical proof of death, with no body, and no death rituals, there is no resolution of grief and long-term closure [11]. The families are stuck in limbo and cling to the optimism that the missing relative may still be alive. The recovery of the bodies or bones of their precious ones not only provides families with internal peace but can also be an important sign of reckoning [12]; it can be a crucial element of the healing process for families and communities. Also, the funeral rituals performed by the family and friends together re-affirm that the person has died. The family may overcome the stage of denial and accept that the person has died. A Nepalese family took an alternative step after the demise of their loved one. They gathered the charred earth from the crash site to help remember their loved one. On reaching Nepal, they completed the last rites according to the Hindu culture. Though there was no body, a dummy was made, and cremation was undertaken.

During incidents like this Ethiopian airline crash where there may be limited chances of the recovery of the remains, the families should be thoroughly informed on the possibilities and permitted to make the decisions that they feel are best, without others telling them what they should do. In unlikely situations, which do not allow a return of identified remains, all information should be passed on to the family members. This can allow them an opportunity to complete the last rites according to their culture in timely manner. This may bring closure to the families who have lost their loved ones in any conflict or disaster.
